# Comparing efficacy and safety of upfront treatment strategies for anaplastic lymphoma kinase-positive non-small cell lung cancer: a network meta-analysis

**DOI:** 10.37349/etat.2023.00187

**Published:** 2023-12-01

**Authors:** Marco Filetti, Pasquale Lombardi, Rosa Falcone, Raffaele Giusti, Diana Giannarelli, Antonella Carcagnì, Valeria Altamura, Giovanni Scambia, Gennaro Daniele

**Affiliations:** Emory University, USA; ^1^Phase 1 Unit, Fondazione Policlinico Universitario A. Gemelli IRCCS, 00168 Rome, Italy; ^2^Department of Experimental Medicine, Sapienza University of Rome, 00168 Rome, Italy; ^3^Medical Oncology Unit, Sant’Andrea Hospital of Rome, 00168 Rome, Italy; ^4^Biostatistics Unit, Scientific Directorate, Fondazione Policlinico Universitario A. Gemelli IRCCS, 00168 Rome, Italy; ^5^Scientific Directorate, Fondazione Policlinico Universitario A. Gemelli IRCCS, 00168 Rome, Italy; ^6^Department of Life Science and Public Health, Università Cattolica del Sacro Cuore, 00168 Rome, Italy

**Keywords:** Non-small cell lung cancer, targeted therapy, anaplastic lymphoma kinase translocations

## Abstract

**Aim:**

This article is based on our previous research, which was presented as a post at the Congress Aiom 2022 Congress and published in *Tumori Journal* as Conference Abstract (*Tumori J*. 2022;108:1–194. doi: 10.1177/03008916221114500). In this paper, a comprehensive presentation of all the achieved results is provided. Several tyrosine kinase inhibitors (TKIs) have been investigated to treat patients with anaplastic lymphoma kinase (ALK)-positive non-small cell lung cancer (NSCLC). However, direct comparisons between these TKIs are lacking, with many only being compared to crizotinib. To address this gap, a network meta-analysis was conducted to compare the efficacy and safety of various first-line systemic therapies for ALK-positive NSCLC.

**Methods:**

A thorough search of PubMed, Embase, and Cochrane Library was performed to identify randomized controlled trials (RCTs) published between January 01, 2000 and April 01, 2022, and included trials that investigated upfront treatments for this molecular subgroup and reported overall survival (OS), progression-free survival (PFS), objective response rate (ORR), and adverse events (AEs) of grade 3 or higher (grade ≥ 3 AEs).

**Results:**

The analysis included 9 RCTs with 2,443 patients receiving eight different treatments: alectinib (at two different dosages), brigatinib, ceritinib, crizotinib, ensartinib, lorlatinib, and chemotherapy. Second and third-generation TKIs significantly prolonged PFS compared to crizotinib, with lorlatinib having the highest probability of yielding the most favorable PFS, followed by alectinib (300 mg or 600 mg). However, only alectinib has been shown to significantly prolong OS compared to crizotinib to date. Lorlatinib appears superior in reducing the risk of central nervous system (CNS) progression, followed by alectinib 600 mg. Ceritinib had the highest rate of AEs, followed by lorlatinib and brigatinib.

**Conclusions:**

Based on the network meta-analysis, alectinib and lorlatinib emerged as the most promising upfront treatment options. These treatments provide prolonged disease control while maintaining an acceptable safety profile.

## Introduction

Lung cancer is responsible for a significant number of cancer-related deaths worldwide, affecting both men and women [1]. Non-small cell lung cancer (NSCLC) is the most common type, accounting for about 80% of all cases [2, 3]. Among NSCLC patients, 2–7% show anaplastic lymphoma kinase (ALK) translocations, with the non-squamous subtype being more common [4].

ALK-positive NSCLC is a subgroup of tumours with unique biological and clinical features, usually detected in a younger, non-smoking population with female prevalence. Since crizotinib was approved in 2011 [5, 6], numerous new-generation inhibitors have been successfully introduced into clinical practice [7–13]. However, all new-generation tyrosine kinase inhibitors (TKIs) were compared to crizotinib, and there is no direct comparison between second and third-generation inhibitors establishing the best initial treatment. Although several meta-analyses used directly compared these treatments’ efficacy and safety data, their values are hindered by the absence of direct comparative trial results [14, 15]. Therefore, a network meta-analysis (NMA) approach could overcome these limits by synthesizing direct and indirect comparisons evidence. This study has the purpose of assessing a comprehensive systematic review of all randomized controlled trials (RCTs) investigating ALK inhibitors in a first-line setting for ALK-positive NSCLC and applies an NMA methodology to estimate a ranking regarding overall survival (OS), progression-free survival (PFS), intracranial efficacy, and adverse events (AEs).

## Materials and methods

### Search strategy

This study adheres to the Preferred Reporting Items for Systematic reviews and Meta-Analyses (PRISMA) extension statement for NMA. A completed PRISMA 2020 checklist was utilized to demonstrate the methodology. The PubMed, Embase and Cochrane Library were comprehensively searched to identify relevant papers investigating upfront systemic therapy for ALK-positive NSCLC. The search strategy included specific terms related to NSCLC and ALK inhibitors: (Non-Small Cell Lung Cancer) OR (Non-Small Cell Lung Carcinoma) OR (Non-Small-Cell Lung Carcinoma) OR (Non-small Cell Lung Cancer) OR (Non-Small-Cell Lung Carcinomas) OR (NSCLC) AND [(ALK-positive) OR (ALK inhibitor) OR (lorlatinib) OR (alectinib) OR (brigatinib) OR (crizotinib) OR (ensartinib) OR (ceritinib)] AND [(phase 3) OR (phase III) OR (randomized) OR (clinical trial)].

Phase III RCTs enrolling patients with advanced ALK-positive NSCLC were included in the analysis. The primary outcomes were OS and PFS; the secondary outcomes were central nervous system (CNS), PFS, and AEs. Two investigators (MF and PL) conducted the initial screening based on titles and abstracts, followed by a thorough review of the entire papers for potentially relevant works. Disagreements were resolved through discussions involving all authors.

### Inclusion and exclusion criteria

Inclusion criteria encompassed phase III RCTs investigating first-line systemic therapy for ALK-positive NSCLC (experimental arm) in comparison to crizotinib (control arm). The studies had to report on PFS, OS, CNS PFS, and AEs. Observational studies, second-line treatment trials, case reports, reviews, editorials, and replies from authors were excluded. Additionally, studies involving neoadjuvant or adjuvant use of ALK inhibitors or TKIs, as well as sequential treatments after chemotherapy, were excluded. In cases where multiple publications were available for the same trial, the most recent publication with the latest data was selected for analysis.

### Data extraction

Two independent investigators, PL and MF, conducted the information extraction process from the included articles. The following data were extracted: first author’s name, publication year, accrual period, number of patients per arm, treatment regimen, median age, gender distribution, study design, median OS, median PFS, objective response rate (ORR), encephalic response rate (ERR), CNS median PFS, and AEs. In addition, hazard ratios (HRs) and 95% confidence intervals (CIs) were included for time-to-event outcomes. Any discrepancies in data extraction were resolved through consensus between the authors.

### Risk of bias assessment

The Cochrane Collaboration tool for assessing the risk of bias in randomized trials (Figures S1 and S2) was used independently by two authors (PL and MF) to assess the risk of bias. Any potential disagreements were resolved through consensus among all authors.

### Statistical analyses

To evaluate the effects of multiple treatment comparisons and provide clinical evidence, an NMA approach was used on selected trials. The analysis estimated both direct and indirect effects. The chi-square (*χ*^2^), *I*-square (*I*^2^), and *Q* tests were used to assess preliminary heterogeneity, with significance set at *I*^2^ > 50% or *P* < 0.05. A random effects analysis model was applied regardless of the significance of the heterogeneity test. An independent NMA was performed for each outcome measure, with HRs and their corresponding 95% CI used to estimate treatment effects. Network plots were drawn to highlight relationships between treatments. Treatment ranking was achieved using frequentist P-scores, considered a frequentist version of the surface under the cumulative ranking area (SUCRA), which measures the extent of certainty that a treatment is better than another averaged over all competing treatments. The R software (version 4.2.1) and the netmeta package [16] were used to conduct the network meta-analyses.

## Results

### Characteristics of included trials

**Figure 1:**
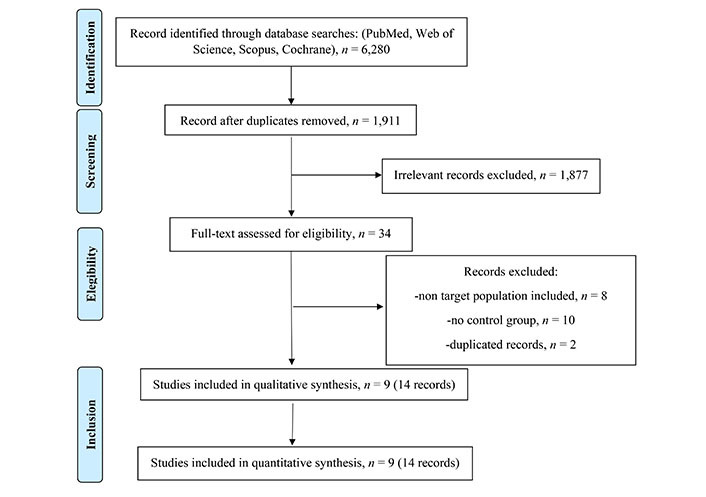
Flow diagram documents the total number of screened, selected, and excluded studies

The literature search initially yielded 6,280 publications, and after removing duplicates, there were 1,911 manuscripts remaining for further analysis. Upon reviewing the titles and abstracts, 34 works were selected for a more detailed reading. After applying the eligibility criteria, 14 papers referred to 9 phase III RCTs were ultimately chosen for inclusion in the study. A summary of the selected studies can be found in Tables S1 and S2, and a flow diagram (Figure 1) documents the total number of screened, selected, and excluded studies. All of the selected studies were RCTs conducted in a naive population, comparing new-generation ALK inhibitors or platinum-based chemotherapy against crizotinib. These 14 articles were published between 2014 and 2021, and involved a total of 2,443 treated patients, with 1,196 patients in the control arm and 1,247 in the experimental arm.

### Treatment comparison

**Figure 2:**
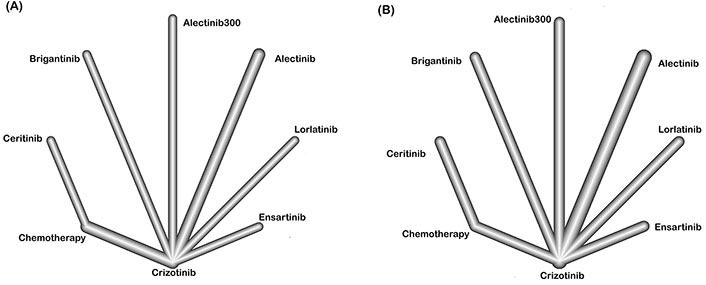
Network of comparisons on PFS (A) and OS (B) in patients with advanced ALK-positive NSCLC

**Figure 3:**
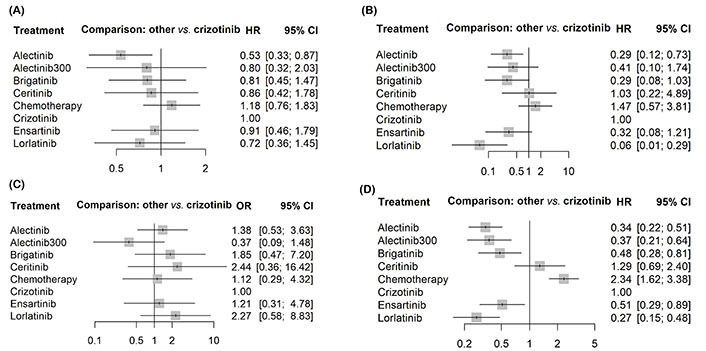
Forest plots showing the association of systemic therapy in metastatic ALK-positive NSCLC. (A) OS; (B) PFS; (C) CNS PFS; (D) grade ≥ 3 AEs

The network of eligible comparisons is represented in Figure 2. Based on available OS and PFS data, the network involved nine trials and seven treatments (crizotinib, ceritinib, alectinib, brigatinib, lorlatinib, ensartinib, and platinum-based chemotherapy). The study reported HR and 95% CIs of all comparisons and considered outcomes in Figure 3A–D.

All the second and third-generation ALK inhibitors, except for ceritinib (HR 1.29, 95% CI 0.69–2.41), displayed a better PFS than crizotinib, with lorlatinib yielding the best benefit (HR 0.28, 95% CI 0.16–0.50). In terms of OS, alectinib consistently provided the best OS benefit (HR 0.53, 95% CI 0.33–0.87); in contrast, no other inhibitors showed significantly better OS than crizotinib.

Lorlatinib showed the most impressive efficacy regarding CNS PFS (HR 0.06, 95% CI 0.01–0.14), followed by alectinib (HR 0.29, 95% CI 0.12–0.73). In contrast, brigatinib, ensartinib, alectinib 300 mg ceritinib, and platinum-based chemotherapy did not show significant superiority over crizotinib in prolonging CNS PFS. No second and third-generation inhibitors show a significantly lower incidence of G3–4 AEs than crizotinib.

### Rank probabilities

The ranking profiles in terms of SUCRA of all comparable treatments were shown in Table 1. Lorlatinib ranked the highest for PFS (P-score 0.96) and encephalic PFS (P-score 0.98), while alectinb has the most significant probability of being the best choice for OS (P-score 0.88) and AEs (P-score 1.00).

**Table 1:** Analysis of the treatment ranking

**Treatments**	**Outcomes of efficacy**						**Outcome of safety**	
	**Rank OS**	**SUCRA**	**Rank PFS**	**SUCRA**	**Rank encephalic PFS**	**SUCRA**	**Rank AEs**	**SUCRA-AEs**
Alectinib	1	88%	2	78%	4	59%	5	55%
Lorlatinib	2	67%	1	96%	1	98%	7	12%
Brigatinib	3	56%	4	55%	2	73%	6	24%
Alectinib 300	4	55%	3	77%	5	56%	1	99%
Ceritinib	5	50%	7	15%	6	21%	8	8%
Ensartinib	6	43%	5	51%	3	68%	4	57%
Crizotinib	7	29%	6	27%	6	21%	2	79%
Platinum-based chemotherapy	8	10%	8	0%	8	2%	3	64%
*I* ^2^	-	39.6%	-	58.7%	-	73.7%	-	-

-: no data

## Discussion

This systematic review and NMA comprehensively analyzed all first-line treatment options’ efficacy and safety data for patients with advanced ALK-positive NSCLC. Several network meta-analyses have been conducted recently [17–20]; however, the NMA provides the most up-to-date picture of the currently available data. Based on SUCRA, treatment ranking showed that lorlatinib had the highest probability of being the best treatment in terms of PFS and encephalic PFS; the study involved 296 patients with ALK-positive NSCLC treatment naive, found that lorlatinib was more effective than crizotinib in preventing the disease progression [10]. This finding is consistent with a previous 2021 meta-analysis from Wang et al. [19], where lorlatinib produced a significant PFS advantage over brigatinib for previously untreated patients with ALK-positive advanced NSCLC. Among the six ALK inhibitors evaluated lorlatinib had the highest probability (96%) of achieving the longest PFS. Based on this data, alectinib appeared to have the best chance of enhancing OS at the time of our analysis. However, this study is merely speculative and only partially addresses the clinical problem resulting from the concurrent approval of various similar treatment options without any direct comparison.

According to Wang et al. [19], the lack of head-to-head comparison among lorlatinib, alectinib, brigatinib, and other TKIs for patients with ALK inhibitor—naive or untreated (ALK inhibitor-naive and chemotherapy-naive) ALK-positive advanced NSCLC, the optimal option for these patients remains undefined [21]. Thus, well-designed comparative trials are required to validate the findings of this study. When approaching this type of analysis, it is essential to underline that the maturity of the follow-up data can radically change ranking at each update in tumors with such prolonged median OS [22]. Furthermore, the possibility of crossover in disease progression may influence OS data. Probably, a more extended observation will demonstrate a significant advantage in OS for second-and third-generation TKIs such as brigatinib, ensartinib, and lorlatinib.

Drug-related AEs are critical issues to consider when choosing the initial therapeutic approach. The youthful ALK-positive demographic is anticipated to undergo prolonged therapeutic regimens over numerous years. Consequently, evaluating the quality of life becomes imperative as a co-primary focus in all RCTs within this context. Disregarding the substantial potential for markedly improved therapies to positively influence patients’ quality of life would be a significant oversight. Notably, lorlatinib has exhibited remarkable efficacy in addressing ALK-positive NSCLC; however, it is important to acknowledge its distinct adverse effect profile [23, 24]. Studies have reported hyperlipidemia as a typical AE, with many patients requiring statin therapy introduction or titration [24]. Mild changes in mental status have also been reported, though they were generally mild and improved or resolved upon dose interruptions or reductions in phase I–II studies [24]. A broad range of CNS side effects has also been described, including changes in cognitive function (such as memory impairment, confusion, and disturbances in attention), mood (such as irritability, anxiety, depression, flat affect, and euphoria/mania), and speech (such as slowed speech and difficulty in word finding) [24]. Tailoring treatment to each patient (usually young and still working) will require deep knowledge and consideration of lifestyle and activity. At least two more aspects should be considered in approaching the ALK-positive NSCLC. The first regards the choice of the first line in consideration of further lines (due to the resistance mechanisms) [25, 26]. The second one is more related to the pharmaco-economics aspects of the treatment choice [27]. Regarding the second, this study deliberately does not face this particular aspect of the question and should be included in all further research in this field.

Regarding the first question about the sequence of treatments for NSCLC patients with ALK translocation, the available data remains insufficient. Typically, this information is derived from retrospective or even preclinical data, relying on the occurrence frequency of molecular resistance mechanisms [28, 29]. Based on both these mechanistic and practical considerations, lorlatinib is the only drug that does not permit crossing to another ALK inhibitor at the progression disease (PD) [30], while most patients progressing on all the other drugs could benefit from lorlatinib. Generally, the best sequence strategy should consider systemic activity, CNS activity, ALK variants, mechanisms of resistance, and toxicity profile [31]. In conclusion, among the currently developed ALK inhibitors, lorlatinib provides the highest probability for the best NSCLC control (both overall and for the CNS metastases), while the best OS and toxicity profile are up to alectinib.

## Abbreviations

AEs: adverse events
ALK: anaplastic lymphoma kinase
CIs: confidence intervals
CNS: central nervous system
HRs: hazard ratios
NMA: network meta-analysis
NSCLC: non-small cell lung cancer
OS: overall survival
PFS: progression-free survival
RCTs: randomized controlled trials
SUCRA: surface under the cumulative ranking area
TKIs: tyrosine kinase inhibitors

## Supplementary materials

The supplementary material for this article is available at: https://www.explorationpub.com/uploads/Article/file/1002187_sup_1.pdf.

## Declarations

### Availability of data and materials

Study data made available upon reasonable request to the first author (Marco Filetti, marco.filetti@policlinicogemelli.it).

### Copyright

© The Author(s) 2023.
