# Supplementary material for: Comparing efficacy and safety of upfront treatment strategies for anaplastic lymphoma kinase-positive non-small cell lung cancer: a network meta-analysis
Source: Explor Target Antitumor Ther. 2023 Dec 1;4(6):1136–44. doi: 10.37349/etat.2023.00187 (PMC10784113; doi:10.37349/etat.2023.00187)
Supplement: Supplementary file 1 [file 1002187_sup_1.pdf]

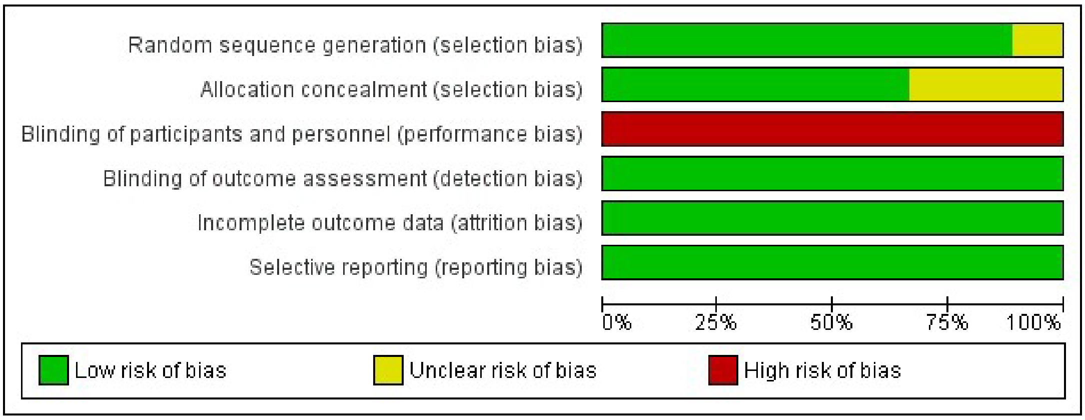

Figure S1. Risk of bias graph: review authors' judgements about each risk of bias item presented as percentages across all included studies

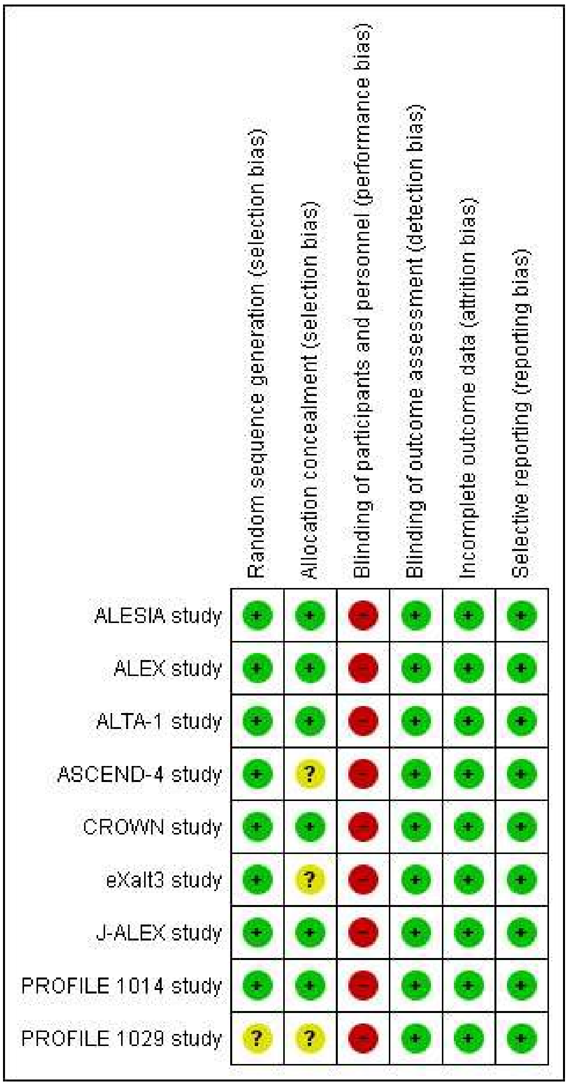

Figure S2. Risk of bias summary: review authors' judgements about each risk of bias item for each included study. Green legend: low risk of bias; yellow legend: unclear risk of bias, red legend: high risk of bias

Table S1. Summary of the included studies

Study	Year	Updated results	Primary endpoint	Number of patients (treatment)	Number of patients (control)	Treatment	Control
**Study**	2014	2019	BICR-PFS	172	171	Crizotinib 250 mg BID	Cisplatin 75 mg/m^2 or carboplatin AUC 5–6 plus pemetrexed 500 mg/m^2 q21
**PROFILE 1014 [6, A]**	2017	NA	BICR-PFS	189	187	Ceritinib 750 mg	Cisplatin 75 mg/m^2 or carboplatin AUC 5–6 plus pemetrexed 500 mg/m^2 q21
**ASCEND-4 [11]**	2017	2020	BICR-PFS	103	104	Alectinib 300 mg BID	Crizotinib 250 mg BID
**J-ALEX [9, B]**	2017	2020	Investigator-assessed PFS	152	151	Alectinib 600 mg BID	Crizotinib 250 mg BID
**ALEX [12, 22]**	2018	NA	BICR-PFS	77	89	Crizotinib 250 mg BID	Cisplatin 75 mg/m^2 or carboplatin AUC 5–6 plus pemetrexed 500 mg/m^2 q21
**PROFILE 1029 [C]**	2020	2022	BICR-PFS	149	147	Lorlatinib 100 mg	Crizotinib 250 mg BID
**CROWN [10, D]**	2019	NA	Investigator-assessed PFS	125	62	Alectinib 600 mg BID	Crizotinib 250 mg BID
**ALESIA [8]**	2018	2021	BICR-PFS	137	138	Brigatinib 180 mg	Crizotinib 250 mg BID
**ALTA-1 [E, F]**	2021	NA	BICR-PFS	143	147	Ensartinib 225 mg	Crizotinib 250 mg BID

NA: not applicable

Table S2. Results of studies included

Study	Follow up	Year	Median Follow Up	Progression free survival	Overall survival
**PROFILE 1014 [6, A]**	**First publication**	2014	16.7 months and 17.4 months, respectively	7 *vs* 10.9, HR 0.45 [95% CI, 0.35 to 0.4]; *P*<0.001	NR *vs* NR HR 0.82 [95% CI, 0.54 to 1.26]; *P*<0.36
	**Updated results**	2018	45.5 months and 45.7 months, respectively	NA	47.5 *vs* NR HR 0.760 [95% CI, 0.548 to 1.053]; *P*<0.0978
**ASCEND-4 [11]**	**First publication**	2017	19.7 months in both arms	8.1 *vs* 16.6, HR 0 .55 [95% CI, 0.42 to 0.73]; *P* < 0.001	26.2 *vs* NR HR 0.73 [95% CI, 0.5 to 1.08]; *P* < 0.056
	**Updated results**	NA	NA	NA	NA
**J-ALEX [9, B]**	**First publication**	2017	12.2 months and 12 months, respectively	10.2 *vs* NR, HR 0 .34 [95% CI, 0.17 to 0.71]; *P* < 0.0001	NA
	**Updated results**	2020	42.2 months and 42.4 months, respectively	10.2 *vs* 34.1, HR 0 .37 [95% CI, 0.26 to 0.52];	43.7 *vs* NR HR 0.8 [95% CI, 0.35 to 1.82];
**ALEX [12, 22]**	**First publication**	2017	17.6 months and 18.36 months, respectively	11.1 *vs* NR, HR 0 .47 [95% CI, 0.34 to 0.65]; *P* < 0.001	NR *vs* NR HR 0.76 [95% CI, 0.48 to 1.2]; *P* < 0.24
	**Updated results**	2020	46 months in both arms	10.9 *vs* 34.8, HR 0 .43 [95% CI, 0.32 to 0.58]; *P* < 0.0001	57.4 *vs* NR HR 0.67 [95% CI, 0.46 to 0.98]; *P* < 0.0376
**PROFILE 1029 [C]**	**First publication**	2018	21.6 months and 22.5 months, respectively	6.8 *vs* 11.1, HR 0 .40.2 [95% CI, 0.286 to 0.565]; *P* < 0.0001	27.7 *vs* 28.5 HR 0.879 [95% CI, 0.556 to 1.445]; *P* < 0.327
	**Updated**	NA	NA	NA	NA

	**results**				
**CROWN [10, D]**	**First publication**	2020	14.8 months and 18.3 months, respectively	9.3 *vs* NR, HR 0 .28 [95% CI, 0.19 to 0.41]; *P* < 0.001	NR *vs* NR HR 0.72 [95% CI, 0.41 to 1.25]; *P* n.s.
	**Updated results**	2022	29.3 months and 36.7 months, respectively	9.3 *vs* NR, HR 0 .27 [95% CI, 0.18 to 0.39];	NA
**ALESIA [8]**	**First publication**	2019	15 months and 16.2 months, respectively	11.1 *vs* NR, HR 0 .22 [95% CI, 0.13 to 0.38]; *P* < 0.0001	NR *vs* NR HR 0.28 [95% CI, 0.12 to 068];
	**Updated results**	NA	NA	NA	NA
**ALTA-1 [E, F]**	**First publication**	2018	9.3 months and 11 months, respectively	9.8 *vs* NR, HR 0 .49 [95% CI, 0.33 to 0.74]; *P* < 0.001	NR *vs* NR HR 0.98 [95% CI, 0.5 to 1.93];
	**Updated results**	2021	15.2 months and 40.4 months, respectively	11.1 *vs* 40.4, HR 0 .48 [95% CI, 0.35 to 0.66]; *P* < 0.0001	NR *vs* NR HR 0.81 [95% CI, 0.53 to 1.22];
**eXalt3 [13]**	**First publication**	2021	20.2 months and 23.8 months, respectively	12.7 vs 25.8, HR 0 .51 [95% CI, 0.35 to 0.72]; *P* < 0.0001	NR vs NR HR 0.91 [95% CI, 0.54 to 1.54]; *P* = 0.73
	**Updated results**	NA	NA	NA	NA

NA: not applicable

References of supplementary materials

A. Solomon BJ, Kim DW, Wu YL, Nakagawa K, Mekhail T, Felip E, et al. Final overall survival analysis from a study comparing first-line crizotinib *versus* chemotherapy in ALK-mutation-positive non-small-cell lung cancer. J Clin Oncol. 2018;36:2251–8.

B. Nakagawa K, Hida T, Nokihara H, Morise M, Azuma K, Kim YH, et al. Final progression-free survival results from the J-ALEX study of alectinib *versus* crizotinib in *ALK*-positive non-small-cell lung cancer. Lung Cancer. 2020;139:195–9.

C. Wu YL, Lu S, Lu Y, Zhou J, Shi YK, Sriuranpong V, et al. Results of PROFILE 1029, a phase III comparison of first-line crizotinib *versus* chemotherapy in East Asian patients with *ALK*-positive advanced non-small cell lung cancer. J Thorac Oncol. 2018:13:1539–48.

D. Qing Z, Kim HR, Soo RA, Chiu CH, Hayashi H, Kim SW, et al. 992P Updated analyses from the CROWN study of first-line lorlatinib *vs* crizotinib in Asian patients with ALK-positive non-small cell lung cancer (NSCLC). Ann Oncol. 2022;33:S1007.

E. Camidge DR, Kim HR, Ahn MJ, Yang JCH, Han JY, Hochmair MJ, et al. Brigatinib *versus* crizotinib in ALK inhibitor-naive advanced ALK-positive NSCLC: final results of phase 3 ALTA-1L trial. J Thorac Oncol. 2021;16:2091–108. Erratum in: J Thorac Oncol. 2022;[Epub ahead of print].

F. Camidge DR, Kim HR, Ahn MJ, Yang JC, Han JY, Lee JS, et al. Brigatinib *versus* crizotinib in *ALK*-positive non-small-cell lung cancer. N Engl J Med. 2018;379:2027–39.
